# Supplementary material for: A set of multi-entry identification keys to African frugivorous flies (Diptera, Tephritidae)
Source: Zookeys. 2014 Jul 24;(428):97–108. doi: 10.3897/zookeys.428.7366 (PMC4143993; doi:10.3897/zookeys.428.7366)
Supplement: Supplementary material 6 — Key to Ceratitis [file zookeys-428-097-s006.zip › SF6_ZooKeys_key to Ceratitis/key/SF6_ZooKeys_key to Ceratitis/Media/Html/Ceratitis stictica .htm]

Ceratitis (Ceratalaspis) stictica Bezzi


***Ceratitis*** **(*Ceratalaspis*)
*stictica* Bezzi**

*Ceratitis stictica* Bezzi, 1909: 277.

Body
length: 4.93 (4.70-5.30) mm; wing length: 4.93 (4.50-5.20) mm.

Male

Head.
Antenna yellow-orange. Third antennal segment three times as long as second
segment. Arista with medium long hairs over entire length. Frons yellow-orange,
with short scattered hairs which are largely the same colour as frons; convex,
in lateral view not distinctly projecting forwards at antennal implant.
Chaetotaxy normal for subgenus. Frons and face sometimes with brownish median
spots, no distinct bands.

Thorax.
Postpronotum yellow-white, spotted. Ground colour of mesonotum pale with
yellow-orange to orange tinge. Chaetotaxy normal for subgenus. Scapular setae
pale (occasionally more darkish). One anepisternal bristle. Scutellum white
basally, otherwise yellow with three black separate markings apically; basally
with two dark separate spots. Subscutellum with three separate spots, sometimes
joined dorsally.

Legs
yellow; setation typical for subgenus, mainly pale especially on femora.
Posterior and posterodorsal rows on fore femur pale. Ventral spines on fore
femur with pale bristles, occasionally dark.

Wing
bands with extensively yellow markings. Banding variable, marginal band
continuous; cubital band usually weakly joined with discal band, sometimes
free; medial band indistinct and/or free, sometimes joined with marginal band;
crossvein r-m at middle of discal cell. Crossvein dm-cu posteriorly more
inwards than anteriorly.

Abdomen.
Yellow. Setation and banding typical for subgenus.

Female.

As
male except for the following� characters: Subscutellum sometimes completely
black. Oviscape shorter than abdominal terga 3-6 combined.

(Description
after De Meyer, 1998)
